# Space Microgravity Alters Neural Stem Cell Division: Implications for Brain Cancer Research on Earth and in Space

**DOI:** 10.3390/ijms232214320

**Published:** 2022-11-18

**Authors:** Sophia Shaka, Nicolas Carpo, Victoria Tran, Carlos Cepeda, Araceli Espinosa-Jeffrey

**Affiliations:** Department of Psychiatry, Semel Institute for Neuroscience and Human Behavior, The University of California Los Angeles, Los Angeles, CA 90095, USA

**Keywords:** human neural stem cells, abnormal cell division, cytokinesis, gravitational research, brain, microgravity, space flight

## Abstract

Considering the imminence of long-term space travel, it is necessary to investigate the impact of space microgravity (SPC-µG) in order to determine if this environment has consequences on the astronauts’ health, in particular, neural and cognitive functions. Neural stem cells (NSCs) are the basis for the regeneration of the central nervous system (CNS) cell populations and learning how weightlessness impacts NSCs in health and disease provides a critical tool for the potential mitigation of specific mechanisms leading to neurological disorders. In previous studies, we found that exposure to SPC-µG resulted in enhanced proliferation, a shortened cell cycle, and a larger cell diameter of NSCs compared to control cells. Here, we report the frequent occurrence of abnormal cell division (ACD) including incomplete cell division (ICD), where cytokinesis is not successfully completed, and multi-daughter cell division (MDCD) of NSCs following SPC-µG as well as secretome exposure compared to ground control (1G) NSCs. These findings provide new insights into the potential health implications of space travel and have far-reaching implications for understanding the mechanisms leading to the deleterious effects of long-term space travel as well as potential carcinogenic susceptibility. Knowledge of these mechanisms could help to develop preventive or corrective measures for successful long-term SPC-µG exposure.

## 1. Introduction

To determine the health risks associated with long-term space travel, it is essential to consider the effects of space microgravity (SPC-µG) on the brain. It is well-established that astronauts oftentimes experience adverse effects following a visit to outer space including the National Aeronautics and Space Administration (NASA)-recognized spaceflight-associated neuro-ocular syndrome (SANS) [1]. Potential risk factors as well as limitations to lengthy space missions and stays at the International Space Station (ISS) are posed by SANS, among other difficulties that astronauts face due to the gravitational differences in space. This is particularly important because issues such as SANS can be experienced after as little as 34 days, and unusual pathologic neuro-ophthalmic findings have been shown after both short- and long-term space travel [2].

Many studies on the use of microgravity have been performed using an array of cells both committed to a specific phenotype depending on the organ of study as well as uncommitted cells known as stem cells. The origin of stem cells includes murine and human origin. Among these are adult stem cells, embryonic stem cells, induced pluripotent stem cells (iPS), and organ-specific stem cells [3]. Recent publications have described changes in stem cells when exposed to simulated or space microgravity. Nonetheless, there is limited information in what concerns neural stem cells. Prior research looking at the effects of microgravity on neural stem cells has primarily been conducted with the use of simulated microgravity (sim-µG). Sim-µG studies use machines that are able to simulate low gravity conditions such as the Mitsubishi 3D-Clinostat [4]. The use of different microgravity platforms with application on stem cells and differentiated cells has recently been reviewed [3]. Only a few studies have shown the effect of sim-µG or SPC-µG specifically on neural stem cells. In this regard, Silvano and collaborators have shown that murine cerebellar neural stem cells (NSCs) respond to sim-µG by the arrest of their cell cycle in the S-phase with a concomitant increase in apoptosis. These changes occurred in a transient manner because upon return to 1G (Earth’s gravity), the authors described that the cells recovered their stemness and a normal cell cycle [5]. We have previously reported a result that appears to contradict Silvano et al.’s findings, where we found that NSCs in sim-µG proliferated more and displayed a smaller diameter than 1G grown NSCs [6]. A recent discovery that spaceflight upregulated proliferation and survival genes in neural crest derived stem cells while the random positioning machine upregulated differentiation and inflammation genes emphasized the uniqueness of space flight conditions when compared to Earth controls [7].

Our current research avoids the use of sim-µG and moves directly to the source of microgravity -- outer space. SPC-µG has demonstrated more pronounced effects of certain phenomena when compared to sim-µG [8]. Although radiation and other environmental differences cannot be excluded as contributing factors, the findings are similar between sim-µG and SPC-µG, save for the difference in degree [8].

The results of the current study provide outcomes not previously reported in sim-µG studies, with the additional benefit of added relevance to the astronauts’ health. Here, four conditions of NSCs derived from human induced pluripotent stem cells (hiPS) are considered. In the ground control condition (1G), NSCs did not experience any SPC-µG, although other cellular conditions were the same as the experimental groups (more details on the procedure are available in Section 2). The second group consisted of 1G naïve sister cells of the space-flown NSCs that were treated with the secretome, which is our term for the secreted medium from space-flown cells. Finally, we also looked at two groups of NSCs following space flight, with the first group captured one week post-flight, and the final group captured at two weeks post-flight.

Due to the nature of the study looking at abnormalities in cell division, it is important to understand the intricacies of the cell cycle and the potential stage(s) for error. In the case of one of the abnormalities, we deemed incomplete cell division (ICD) as failed cytokinesis. Cytokinesis is a highly complex, regulated process that requires an extensive number of cellular processes, proteins, and signaling factors to function properly. Cytokinetic failure can occur due to defects in any of its four stages, which include positioning of the division plane, ingression of the cleavage furrow, formation of the midbody, and abscission [9]. Cytokinetic failure following the division of the cell nucleus, or karyokinesis, can result in a tetraploid cell with double nuclei and four copies of each chromosome rather than the expected two. For some cells of the human body such as in the heart or liver, tetraploidy is a normal process [9]. However, in many cases, tetraploidy can be a sign of a deleterious abnormality such as a tumor precursor [10].

Previous work in the literature has confirmed that embryonic and induced pluripotent stem cells behave similarly in terms of cell cycle components. Embryonic stem cell pluripotency is maintained through unique and highly specific signaling. These cells enter the S phase more rapidly with a shortened G1 due to impaired or atypical checkpoint control in embryonic stem cells and unusual activation patterns [11].

Our previous findings showed that NSCs proliferate at a higher rate while in space as well as up to 72 h following space flight, irrespective of flight duration [2,4]. We also showed that changes in the cell diameter occurred post-flight, with 81% of NSCs exhibiting a diameter of 10 µm or higher following space flight compared to 49.2% of the control NSCs [4]. While the undifferentiated NSCs in adult human brain areas such as the subventricular zone and the dentate gyrus of the hippocampus remain quiescent under normal circumstances, the environment of SPC-µG exposure is likely to alter the expected behavior of these cells [12]. The changes described above including increased proliferation and enlarged cell diameter [13] could contribute to symptoms of SANS that astronauts experience post-flight such as intracranial hypertension. Here, in addition to the aforementioned differences, we noticed that some SPC-µG NSCs appeared to have difficulties with the completion of cytokinesis with enough frequency to be of interest for further investigation. The current study takes a closer look at proliferation abnormalities in single cell division. Throughout this paper, the term ICD refers to an ultimate failure of cytokinesis, regardless of whether karyokinesis has occurred, and multi-daughter cell division (MDCD) refers to the appearance of more than two daughter cells as would be typical for a single cell division following cytokinesis. The term abnormal cell division (ACD) refers to either phenomena, ICD and/or MDCD. We investigated the hypothesis that, compared to 1G NSCs, there are significantly increased instances of ICD, MDCD, and ACD following exposure to SPC-µG. We also hypothesized that the space-produced secretome would have a similar effect on naïve NSCs.

## 2. Results

### 2.1. Time-Lapse Microscopy Analysis

#### 2.1.1. A Subpopulation of NSCs Back from Space Displayed Incomplete Divisions

**Figure 1:**
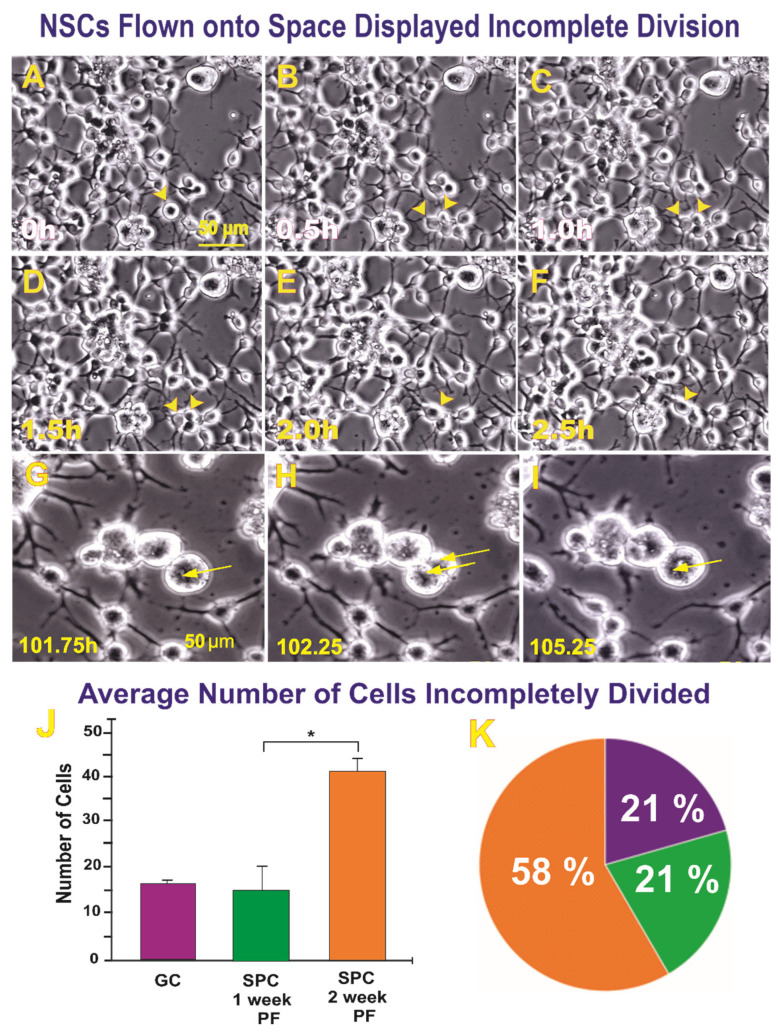
An example from time-lapse microscopy of SPC-NSCs 1 week (**A**–**F**) and 2 weeks after space flight. One week after returning to Earth, the cells had completely recovered from their round trip to space. They were healthy, displaying cell processes, and were proliferative. (**A**) Nonetheless, a subpopulation appeared to have acquired a larger size (arrow) when observed under phase-contrast microscopy. (**B**) When time-lapse images were examined, we found that some cells had started the proliferation process (i.e., the cells appeared to be dividing (arrows)). (**C**,**D**) The cell appeared to have reached the full division process. (**E**) Instead of two daughter cells, they had fused together, (**F**) remaining that way for the rest of the time the cells were imaged. This event lasted 2.5 h (arrowheads show the cells of interest). (**G**,**H**), another example of NSCs two weeks post-flight exhibiting ICD occurring at 102.25 h into time-lapse (shown in part H) and no visible cytokinesis, with the NSC returning to one cell by 105.25 h (shown in part **I**). Cell(s) of interest are indicated by the yellow arrow(s). The cells finally merged and remained that way until the end of the experiment. Scale bar = 50 µm. (**J**) Quantitative data showing that more instances of ICDs were seen in the SPC-exposed NSCs. The average number of cells that exhibited ICD during the 50 h that NSCs were under observation via time-lapse microscopy. The purple bar denotes ground control (1G) NSCs. The green bar represents SPC-NSCs one week after returning from the ISS. The orange bar denotes NSCs two weeks after space flight. Data analysis was performed with one-way ANOVA followed by Tukey multiple comparisons test in which * *p* < 0.05 was defined as statistically significant; and the Chi-square test (for percentages) demonstrated statistically significant differences (*p* = 0.045). Data represent the mean of four separate scenes for all three conditions of NSCs shown. (**K**) This chart represents the average percentage of incomplete division across groups where normal NSCs that remained on Earth and NSCs one week after returning from space did show very similar cell numbers and the same percentage, while two weeks after space flight, the number of ICD cases accounted for 58%.

Observations of SPC-exposed NSCs revealed that although these cells proliferated more than their ground control counterparts, a sub-population of them attempted to divide unsuccessfully. These abnormally dividing cells displayed two cell bodies, yet ultimately did not achieve complete cell division into two daughter cells, instead remaining as a larger cell and preserving that state for the rest of the time-lapse imaging, as shown in Figure 1. In terms of the differences between the 1G and SPC-µG exposed NSCs, Figure 1A–I illustrates an example of incomplete cytokinesis. The average numbers (bar graph) and percentages (pie graphs) show the raw numbers and proportion of cells involved in ICDs throughout the time-lapse videos. There was an upward trend observed among the groups, with the NSCs two weeks after SPC-µG exposure showing the highest number of instances of ICD. For the control group, the average total number of cells was 1606 with an average ratio of 5.67 ICDs per 1606 cells. For the SPC–µG exposed NSCs, there were on average 8.5 ICDs per 1370 NSCs one week after spaceflight, and 11 ICDs per 903 cells across the different scenes analyzed two weeks post-flight.

However, because the total number of cells differed among the three conditions, we also estimated the relative proportions of ICDs. This was calculated by dividing the number of ICDs by the total number of cells present at the exact middle of each time-lapse video. The middle frame represented an approximate mean between the number of cells present at the start of the time-lapse compared with the number of cells present at the end of the video after proliferation had occurred. When comparing the different proportions, the Chi-square test demonstrated statistically significant differences (*p* = 0.045) (Figure 1K).

#### 2.1.2. A Subpopulation of SPC-NSCs Displayed Multi-Daughter Cell Divisions

**Figure 2:**
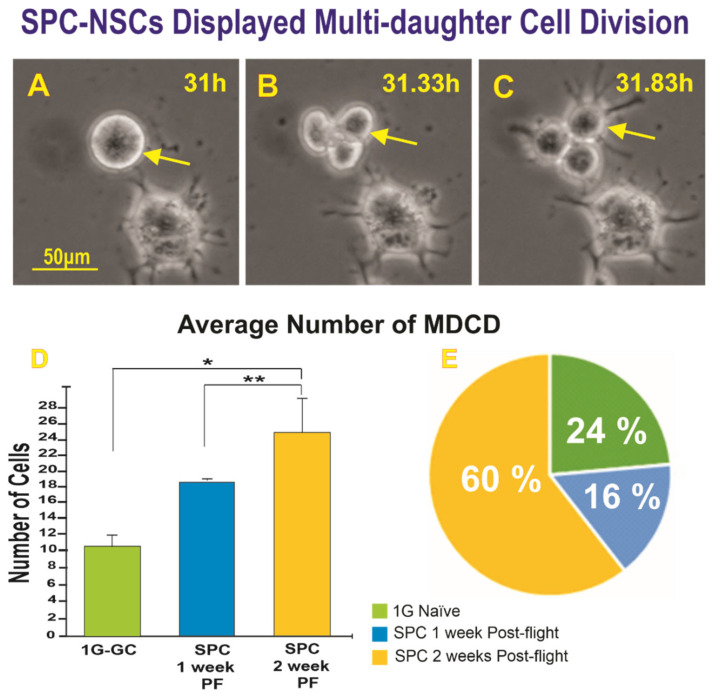
An example from time-lapse microscopy of SPC-µG exposed NSCs exhibiting MDCD 2 weeks post-flight. (**A**) Cells prior to dividing. (**B**) Three hours later, at 31.33 h, there were three cells visible. (**C**) These three daughter cells survived for the duration of the experiment and within 50 min, they had developed cell processes. The yellow arrow in all three images points to the mother cell. Quantitative data where NSCs flown to space displayed more instances of MDCDs were seen one week post-space flight, and even more NSCs presented this behavior. (**D**) The bar graph on the left, shows the average number of cells that exhibited MDCD during the 50 h that the NSCs were under observation via time-lapse microscopy. The green bar denotes ground control (1G) NSCs. The blue bar represents SPC-NSCs one week after returning from the ISS. The yellow bar denotes NSCs two weeks after space flight. (**E**) Results shown as percentage. Data analysis was performed with one-way ANOVA followed by the Tukey multiple comparisons test in which * *p* < 0.05 was defined as statistically significant * *p* < 0.05; ** *p* < 0.001 and the Chi-square test (for percentages) demonstrated statistically significant differences (*p* = 0.045). Please also see MP41 in the Supplementary Materials.

In addition to ICD, we found multi-daughter cell division, which was also examined. An example of multi-daughter cell division where one cell gave rise to three cells at the end of cytokinesis is shown in Figure 2A–C. The average number of cells exhibiting MDCD one week and two weeks after space flight and the percentage of MDCD instances are shown (Figure 2D,E).

**Figure 3:**
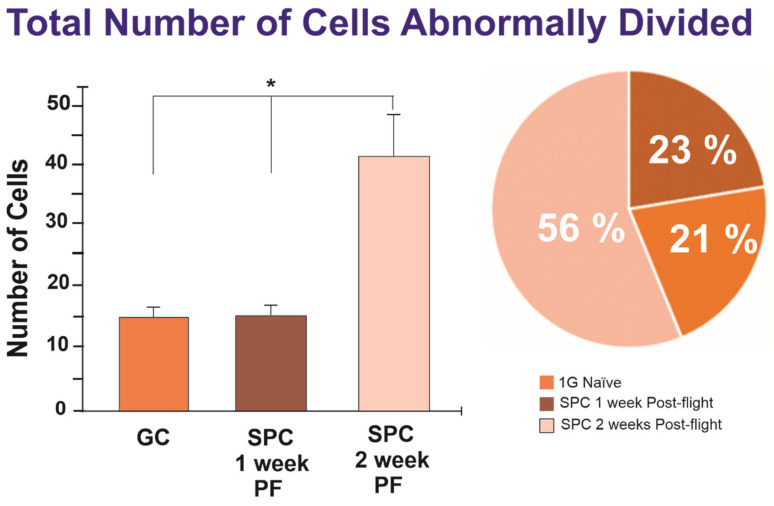
The bar graph on the left depicts the total number of cells abnormally divided (exhibiting either ICD or MDCD) across four scenes of time-lapse microscopy for each of the three groups. The experimental group of SPC-µG exposed NSCs 2 weeks post-flight displayed a statistically significantly greater amount of ACD compared to the control group and the one week post-flight SPC-flown NSCs (*p* = 0.0048 for 1G vs. SPC 2 weeks PF; *p* = 0.0052 for SPC 1 week PF vs. SPC 2 weeks PF). The pie graph shows the difference in the proportion of ICD between the 1G control NSCs (orange), SPC-µG exposed NSCs one week post-flight (brown), and SPC-µG exposed NSCs two weeks post-flight (salmon). NSCs two weeks following space flight displayed a significantly increased frequency of ICD. Data analysis was performed with one-way ANOVA followed by the Tukey multiple comparisons test in which * *p* < 0.05 was defined as statistically significant; and the Chi-square test (for percentages) results were significant with *p* = 0.0135. Data represent the mean of four separate scenes for each color-coded condition.

As both ICD and MDCD (triple divisions) can be considered within the realm of ACDs, we also compared the total number of ACDs among the three conditions. Although the averages differed between the groups in terms of MDCDs, the sum of ICDs and MDCDs proved statistically significant. (Figure 3).

### 2.2. Effects of the Secretome on Naïve NSCs Cell Divisions

**Figure 4:**
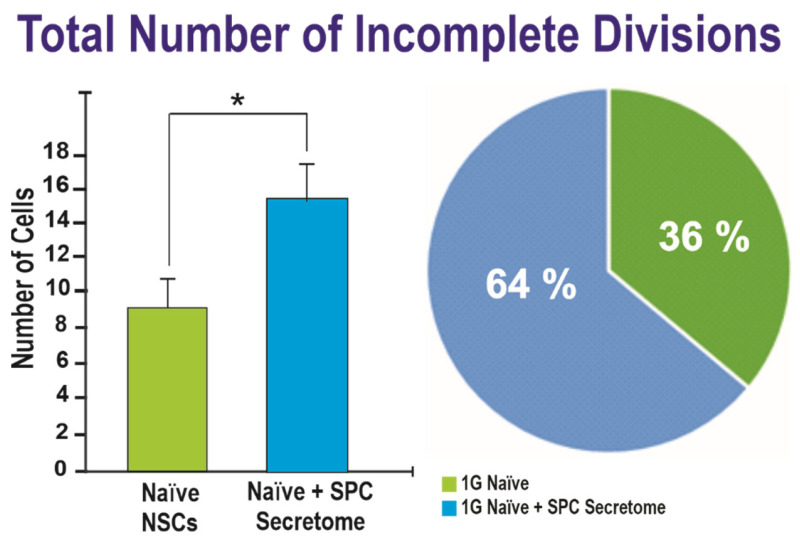
Average number of cells that exhibited ICD during the 70 to 130 h that NSCs were under observation via time-lapse microscopy. The pink bar denotes naïve NSCs with no exposure to SPC-µG or secretome. The turquoise blue bar denotes naïve 1G NSCs following the addition of secretome (*p* = 0.037). The pie chart shows the ratio of incomplete division events compared to the total number of cells in the time-lapse for each condition. Data analysis was performed with one-way ANOVA followed by the Tukey multiple comparisons test in which * *p* < 0.05. Data represent the mean of four separate scenes for each color-coded condition. Chi-square test (for percentages) results were significant with *p* = 0.001. Data represent the mean of four separate scenes for each color-coded condition. See also MP42 in the Supplementary Materials.

To ascertain if these changes had been exerted directly on the cells during space flight, we examined if there were molecules that would have been secreted by NSCs under the influence of microgravity. For this experiment we seeded the cells and followed them for 72 h, time at which we added the secretome to the cells in a 2:1 ratio V/V. We found that the secretome produced similar results in terms of incomplete cytokinesis suggesting that secreted molecules rather than mechanical forces are responsible for these effects (Figure 4).

**Figure 5:**
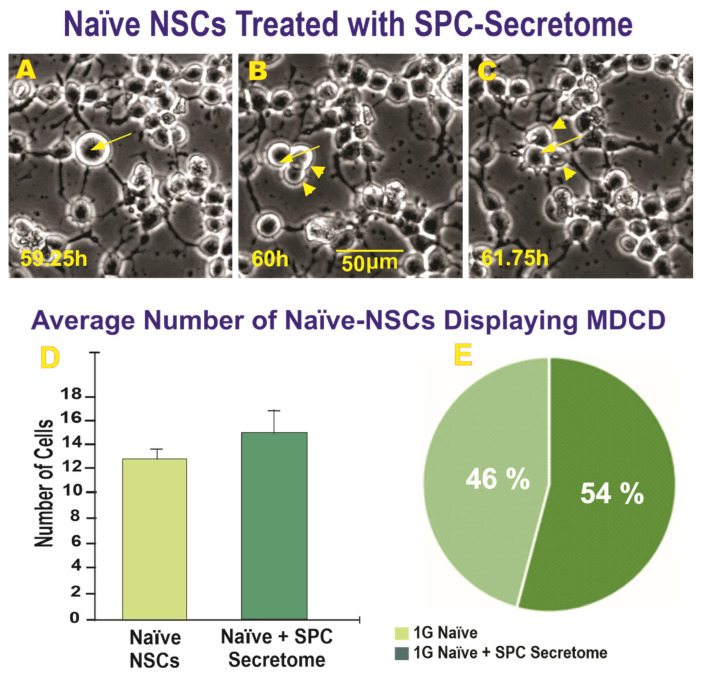
This example from the time-lapse microscopy of naïve 1G NSCs following treatment with SPC-µG NSCs secretome shows: (**A**) A NSC MDCD; (**B**) The cell gives rise to two daughter cells: (**C**) The cells had successfully completed cytokinesis with three cells visible beginning at 60 h and remaining as three cells for the duration of the video. The arrow points to the mother cell and the arrowheads designate the daughter cells. (**D**) The bar graph on the left depicts the total number of cells displaying MDCD across four scenes of time-lapse microscopy for each of the three groups. The secretome produced by NSCs while in space increased the number of triple cell divisions although these differences were not significant. (**E**) The pie graph shows the difference in the proportion of MDCD between the 1G control NSCs. Data analysis was performed with one-way ANOVA followed by the Tukey multiple comparisons and the Chi-square test (for percentages) results were not significant. Data represent the mean of four separate scenes for each color-coded condition.

We examined the question of whether or not the secretome would also produce similar effects on naïve NSCs that had never flown to space in terms of MDCD. An example of MDCD in the naïve NSCs treated with secretome is shown (Figure 5A–C), where one cell divides into three daughter cells and the three cells continue to survive and move around for the duration of the time-lapse. 

**Figure 6:**
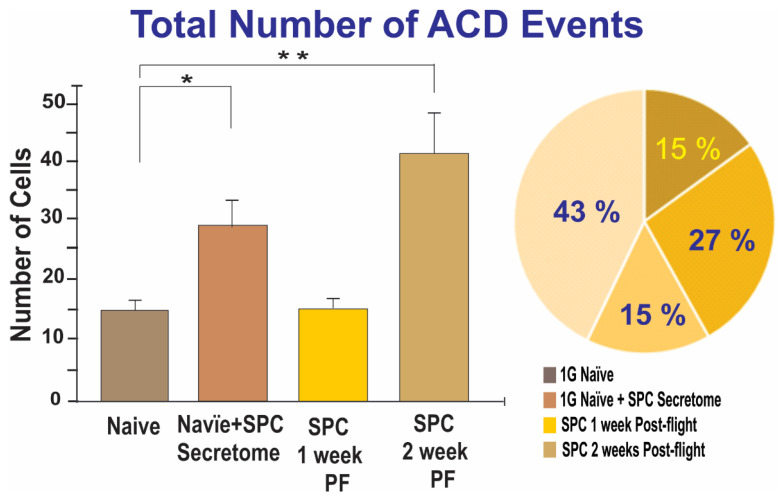
The bars on the graph represent the total number of NSCs abnormally divided (exhibiting either ICD or MDCD) for naïve NSCs, SPC-NSCs, and naïve NSCs treated with SPC-NSC secretome. Data were obtained from four scenes of time-lapse microscopy for each of the four groups. The experimental group of SPC-µG exposed NSCs 2 weeks post-flight (PF) displayed a statistically significantly greater amount of ACD compared to the control group and the one week post-flight SPC-flown NSCs (*p* = 0.0048 for 1G vs. SPC 2 weeks PF; *p* = 0.0052 for SPC 1 week PF vs. SPC 2 weeks PF). Naïve-NSCs results were also significantly different with respect to the naïve (non-treated) NSCs. The pie chart shows the ratio of abnormally divided cells compared to the total number of cells in the time-lapse for each condition. NSCs two weeks following space flight displayed a significantly increased frequency of ACD. Moreover, a significant difference was also found in naïve-NSCs exposed to SPC-secretome. Data analysis was performed with one-way ANOVA followed by the Tukey multiple comparisons test in which * *p* < 0.05 and ** *p* < 0.01 was defined as statistically significant; the Chi-square test (for percentages) demonstrated that the results were significant with *p* = 0.001. Data represent the mean of four separate scenes for each color-coded condition.

While looking at the raw numbers can provide useful information, it is important to consider that the total number of visible cells as filmed through time-lapse microscopy differs for each scene. Therefore, the pie charts in Figure 1 and Figure 3 take this into account in order to display the fraction of total cells that exhibited ICD or ACD. There was a significantly greater proportion of ICDs found in the experimental group of NSCs two weeks post-flight (1.22%) compared to the other groups, with the 1G naïve NSCs at 0.35% and the NSCs one week post-flight exhibiting 0.62% ICD. Concerning the sum of ICD and MDCD, there was also a significantly greater proportion of ACDs found in the SPC-µG exposed NSCs two weeks post-flight (1.68%). The 1G naïve NSCs exhibited ACD at a frequency of 0.58%, the same cells after the addition of secretome exhibited ACD 0.98% of the time, and the SPC-µG exposed NSCs one week post-flight had a percentage of 0.56% the influence of microgravity. In addition to ICD, the raw numbers of cells involved in either ICD or MDCD, deemed ACDs, were also graphed (Figure 6). For the control group, the average total number of cells was 1606 with an average ratio of 9.33 ACDs per 1606 cells. After the medium was switched to incorporate secretome, this value changed drastically, with an average of 15.67 ACDs per 1606 cells. For the SPC–µG exposed NSCs, there were on average 7.63 ACDs per 1370 NSCs one week after spaceflight, while there were on average 15.17 ICDs per 903 cells across the different scenes analyzed two weeks post-flight (Figure 6).

From these data, it is clear that there were statistically significant differences in the amount of ACD as well as ICD alone following cellular exposure to SPC-µG, particularly when looking at NSCs two weeks following SPC-µG exposure. While not always statistically significant, there are also more instances of both ICD and ACD following the addition of secretome to the NSCs.

### 2.3. Immunofluorescence

Immunocytochemistry was performed on both the control and experimental groups of NSCs. These cells were examined for the expression of NSC markers in order to confirm lineage. The normal morphology and physiology of NSCs are partially dependent on the cytoskeleton, which is composed of intermediate filaments, actin, and microtubules.

**Figure 7:**
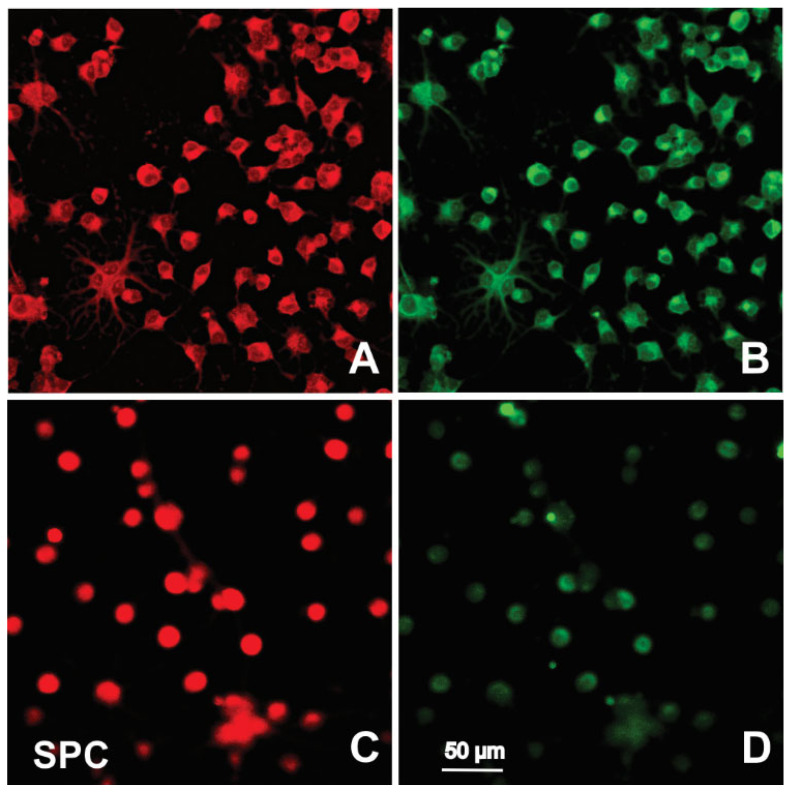
Representative views displaying the immunocytochemical distribution of nestin (red) and tubulin beta II (green). (**A**) Nestin expression in the 1G control NSCs is positive, with nuclei clearly visible. (**B**) Tubulin expression in 1G NSCs is also positive. (**C**) SPC–µG exposed NSCs intensely expressed nestin in most cells. (**D**) Tubulin was faintly expressed in some SPC–µG exposed NSCs but most cells were negative.

One of the proteins detected by immuno-fluorescence was neuroepithelial stem cell protein (nestin). Nestin expression was of interest because as a type VI intermediate filament (IF) protein, nestin is primarily expressed in dividing NSCs. Potential differences in nestin expression between the control and experimental NSCs allows for a deeper examination of morphological differences that may be present after exposure to SPC-µG or secretome. In addition to nestin, tubulin expression was also examined. As a major component protein essential for microtubule formation, tubulin expression is also bound to reveal information regarding the cell cytoskeleton. Furthermore, it has been confirmed that tubulin is expressed in nestin-positive cells, which aids in testing the validity of our work. The results of the immunofluorescence are shown in Figure 7.

## 3. Discussion

It is well-accepted that normal mammalian cell division occurs by the mother cell giving rise to two daughter cells. This is the result of enhanced phosphorylation and dephosphorylation of CDK–cyclin complexes to ensure well-defined transitions through cell cycle stages [13]. Control checkpoints of the cell cycle act as regulators that activate when they detect a “danger” or abnormal situations such as defects in DNA replication or chromosome segregation. Therefore, loss of cell cycle regulation can lead to cancer [14]. We have previously reported a robust enhancement of NSC proliferative potential as well as an increased number of NSCs of larger size [8], a phenomenon also known as hypertrophy, which may occur in response to injury. For example, hypertrophy of cardiac myocytes implicates changes in the gene expression profile where “embryonic genes” reactivate, modifying the contractile protein structure and density of hormone receptors [14]. “Physiologic hypertrophy” is considered adaptive with the goal to improve cell and organ function. Pathologic hypertrophy may be an adaptive response at the beginning, but often results in changes in gene expression that can exacerbate dysfunction and may lead to cancer. Here, we demonstrated that features associated with cancer of the CNS appear to have been accentuated on NSCs by space flight. Through this study, we discovered that a subset of NSCs flown to space displayed hyperplasia, which is characterized by an increased number of daughter cells. Although the percentage of cells showing three or more daughter cells was small, it was statistically significant.

It has previously been shown that when simulating the three-dimensional mechanical confinement found in the in vivo milieu, cancer HeLa cells often divided into three or even five daughter cells [15]. Moreover, higher confinement and stiffness gave rise to increased asymmetry and multi-daughter division [15]. In our case, we did not use confinement nor cancerous cells. However, we found an increased number of instances of abnormal division between one and two weeks post-space flight. We believe that the mechanical forces exerted on NSCs after space flight were those from terrestrial gravity (1G), which makes cells adhere stronger to the plastic substratum that is more rigid than the mesh carrier on which cells grew and flew back and forth to space/Earth. Nonetheless, the results from the naïve NSCs treated with secretome demonstrate that the space environment induces the production and secretion of soluble factors that exert the same behavior on normal self-renewing cells (i.e., naïve NSCs), resulting in ACD without changing the mechanical forces. In terms of MDCD, it is possible that it had resulted as a compensatory mechanism from a stem cell having two or three nuclei trying to remedy multi-nucleated cells by giving three daughter cells in the following generation.

Based on our results, SPC-µG has cellular effects that increase instances of abnormalities in single cell division, particularly when considering ICD and MDCD. The same trend was observed following the addition of the secretome from SPC-NSCs to naïve-NSCs. Thus, elucidation of the molecules that may be responsible for the observed effects is of the essence. Therefore, we analyzed the secretome profile of NSCs flown to space and found that the most enriched molecule was the secreted protein acidic and rich in cysteine (SPARC), produced by these NSCs while in space [16]. Being a matricellular protein, SPARC’s main function is to modulate cell–cell and cell–matrix interactions and it is mainly expressed in the bone and endothelial cells [17,18]. Nonetheless, it is also highly expressed during embryonic life in notochord, chondrocytes, and other tissues of the embryonic skeleton functioning as a remodeling protein [19], and it is also expressed in macrophages at injury sites [20,21,22]. It has been postulated that SPARC may also play a role in different types of cancer, and in some cases, it has promoted the inhibition of breast cancer [23]; SPARC is expressed in grade 3 tumors, but other studies have shown that higher expression of SPARC correlated with decreased metastasis [24]. There is a compilation of studies on the effects of different levels of SPARC expression in tumors and neighboring cells where in some cases SPARC is deleterious and in other types of cancer, it inhibits proliferation and metastasis [19]. For example, a high expression of SPARC has been reported in advanced, metastatic melanoma [25]. In brain gliomas, SPARC is detrimental in that it promotes the survival and invasion of glioma cells [19]. High levels of purified SPARC act as an inhibitor of both neuroblastoma growth and angiogenesis [26,27]. SPARC is considered by some as a cancer therapeutic agent due to its ability to inhibit proliferation and enhance apoptosis in the ovary, colorectum, pancreas, neuroblastoma, and leukemia [19]. However, in the case of neural cells, it is deleterious.

Although our NSCs were not tumor derived, it is possible that NSCs in space display this novel vulnerability that might be triggered and/or enhanced to increase the frequency of abnormal cell division as a function of time. This phenomenon is similar to the progression of a benign tumor into a malignant tumor, and it could be lethal, endangering the space mission. Since our study was performed on Earth’s gravity, where naïve NSCs were not exposed to the mechanical changes between microgravity and 1G, we believe that the secretome produced by SPC-NSCs holds the key for the modulation or downregulation of the abnormal cellular behaviors ICD and MDCD observed.

Having previously seen enhanced proliferation and hypertrophy [8], and in the current study hyperplasia, in our NSCs post-flight, it is easy to speculate that SPARC under different gravity conditions such as on Mars or our Moon will be differentially regulated according to the G-force the cells are subjected to. Therefore, modulation of SPARC’s gene expression may lead us to harness the countermeasures necessary to protect the CNS of the astronauts from neurodegeneration and cancer during and after space flight. These attributes, together with the increase in abnormal cell division, most likely contribute to SANS and other health complications during or following space flight. Stem cells are unique in that they are the only human cell type with the ability to self-renew and produce an unlimited quantity of daughter cells [19]. Therefore, any errors during cell division can lead to the promotion of cancer. Endogenous damage to DNA as well as pathological and environmental factors can lead to such cancer-promoting errors. Chemical and physical signals acting upon stem cells can impact the life and death of the cell as well as their differentiation process. Therefore, the risk of cancer is heavily influenced by extrinsic factors known as the ‘stem cell environment’ or niche [19,20]. As opposed to directly altering DNA, non-genotoxic agents are able to increase the risk of cancer through increasing cell proliferation [21]. The changes observed during cell division noted in this paper have features of malignant stem cells, mimicking the carcinogenic changes hypothesized to lead to cancers [22].

It is known that non-conventional mitosis events lead to tumor progression. Mechanosensing is important for proper cell physiology and tissue homeostasis. Here, we propose that SPARC is a gravity-sensor or gravitometer protein secreted by NSCs in response to an environmental change that is gravity (G). Thus, SPARC’s upregulation by microgravity serves as a marker for us to start designing strategies to regulate this gene, hence regulate proliferation and other cues altered by µG. A factor not studied in our cells was space radiation, which may also play a role in these changes and more work is needed to ascertain its net contribution to potential brain cancer produced by space missions.

We have to acknowledge an important limitation of our current study on abnormalities in stem cell division after exposure to microgravity. A detailed cell cycle analysis with correlation to cytokinesis should be conducted before drawing any conclusions about when during the cell cycle abnormalities are likely to occur. As all the cells returning from space were used for the current experiments, and also due to funding constraints, those studies will be considered as a top priority in the future. Another future direction that this research could explore is a more detailed and high-definition exploration into the daughter cells resulting from MDCD as well as the cells following ICD. Neither the proposal nor funding for this project included the study of the mechanical effects of microgravity on our cells. To the best of our knowledge, there are no published studies on the mechanical properties of non-cancerous NSCs in microgravity and after microgravity exposure. It is known that the cell cytoskeleton has a significant role in adapting cells to mechanical influences such as those caused by gravity changes. In fact, it has been shown that several hours or days of µg exposure leads to significant cytoskeletal changes in the form of microfilament thinning and redistribution [28]. 

Aneuploidy, which is defined as the loss or gain of one or more chromosomes from a diploid gene, is a sign of genomic instability in human cancer cells. Aneuploidy likely represents an early significant event in tumorigenesis [29]. With the current resources, we could not tell if the result of the ACDs could be affecting the cells at a chromosomal level. Therefore, future research could aid the analysis of these cells and the potential detriments of abnormalities in cell division.

In addition, prior research has found that neuroblastoma cancer cells exhibit increased instances of asymmetrical cell division, where one stem cell progenitor and one differentiated cell are the daughter cells produced in a single cell division. This asymmetric division results from a decreased expression of the MYCN proto-oncogene [30]. A future genomics profiling study, looking into the expression of the MYCN gene as well as other proto-oncogenes associated with defects in cell division and proliferation could further research into the potential connection between the abnormalities seen following SPC-µG exposure and cancer cells.

A recent study has shown that in the mouse brain, there are polyploid neurons throughout the development of the neocortex [31]. Nonetheless, it is not clear that the study followed the mice through adulthood. Exit from the cell cycle during development coincides with the differentiation of neurons that is critical for neuronal function. Maintaining a non-dividing state in neurons is critical for brain function [32,33]. When cells exit the cell cycle, they have diploid (2C) DNA content. There is evidence indicating that polyploid cells in the CNS are more prevalent than previously thought and they may be involved in the physiology and pathology of the brain, where cell cycle re-entry is widely deleterious to neurons. To date, the function of mononucleated neurons in the brain or that of binucleated neurons in the autonomic nervous system is not known [34]. It has been hypothesized that these neurons are involved in cell cycle re-entry and neurodegeneration, and neurons entering the cell cycle as well as bi-nucleated neurons have also been reported in Alzheimer’s disease [35,36,37]. Therefore, space-based studies will help humans on Earth and astronauts during and after space missions, as summarized in a recent review [38].

Finally, the literature shows that analysis of the mesenchymal stem cell (MSC)-secretome promotes both NSCs commitment to the oligodendroglial lineage, differentiation, and myelin formation and differentiation of neural stem cells in vivo. The secreted protein tissue inhibitor of metalloproteinase type 1 (TIMP-1) was revealed to be an active component of the MSC-conditioned medium. Nonetheless, this work was performed in 1G, not in microgravity. Moreover, it induced cell commitment and lineage progression, which has nothing to do with abnormal cell division.

It is likely that a vast array of small molecules and proteins including cytokines and other factors are secreted by NSCs on Earth. However, characterization of those factors produced and secreted by NSCs is still in its infancy. Thus far, only three factors (PGE2, TGF-β1, and TGF-β2) have been unequivocally identified. Others are still awaiting identification [39]. For this reason, we did not want to speculate further on the nature of possible molecules that might be responsible for the abnormal cell division we report in this manuscript. To our knowledge, we are the first group studying the effects of the secretome produced in space by hiPS derived NSCs. Analysis of the NSCs-produced secretome is currently being performed and we hope to report the data in the near future.

## 4. Materials and Methods

### 4.1. Space Flight

**Figure 8:**
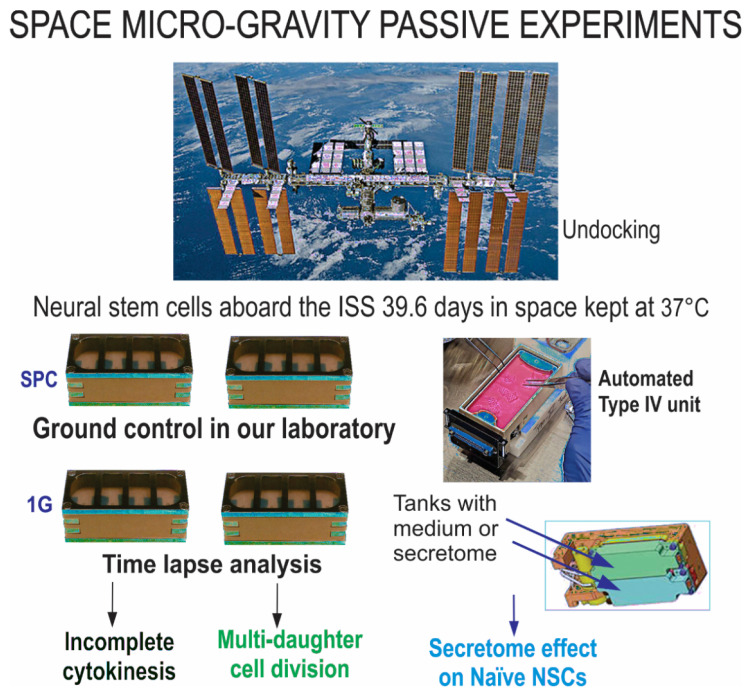
NSCs were seeded on floating mesh carriers that promote strong cell adhesion. Four cell carriers of 2 × 2 mm were placed in each well pre-launch. Cells flew onboard Space X-16, spent a total of 39.6 days in space at the ISS, 248 m (400 km) above the Earth’s surface. Cells were transferred to Long Beach (CA, USA) Airport after splashdown and brought to the UCLA laboratory at 37 °C. Upon arrival, cells were seeded into flasks or flaskettes and allowed to recover for six hours in the incubator and subsequently placed in the imaging system (Adapted from Shaka et al., 2021 [13]).

Induced pluripotent human neural stem cells known as cell line “CS83iCTR-33n21” derived from skin fibroblasts were used for the space flight. The cells were reprogrammed by Cedars-Sinai Medical Center through a material transfer agreement and subsequently induced using the STM medium. For further details regarding the steps of the space flight protocol, see our previous publications as well as Figure 8 below [3]. Both space-flown and ground control cells underwent the same flight preparation procedures, and all cells were grown in hardware and pre-seeded onto mesh carriers in passive units. For this study, both gravity conditions used hardware from Yuri through the payload developer Space Technology and Advanced Research System (STaARS). We used the facility known as the STaARS-EF-1 platform on board the ISS, with flight-tested growth and observation chambers shown in Figure 8. The hardware-units containing the experiment electrically and mechanically interfaced through a clip-in-place mechanism onto the STaARS EF-1.

**Figure 9:**
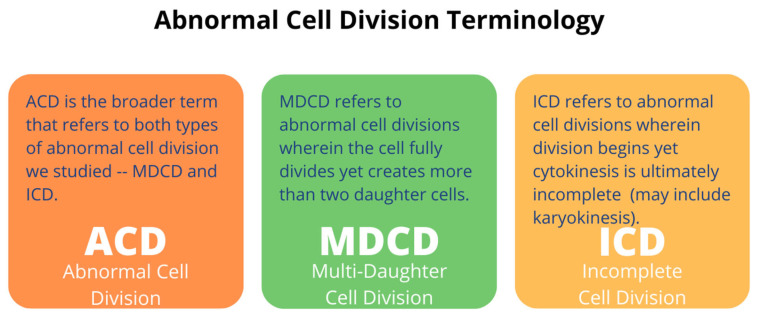
An elaboration upon the specific types of abnormal cell division we defined for the scope of this project. The numbers of abnormally dividing cells were counted by hand through the use of Zeiss ZEN Blue Microscopy software 2020.

SPC-µG exposure: NSCs were flown aboard SpaceX-16 to the ISS and remained onboard the ISS for 39.6 days before returning to Earth, as shown in Figure 8. The cell chambers contained 2 × 2 mm mesh carriers and the culture medium was equilibrated overnight at 5% CO_2_ in the incubator where cells were maintained before the flight. Upon arrival, cells were seeded into flasks or flaskettes and allowed to recover for six hours in the incubator and subsequently placed in the imaging system. (Figure modified from Shaka et al., 2021 [13]). A chart defining the abnormal cell division terminology is also shown (Figure 9).

### 4.2. Secretome Collection and Naïve NSC Cell Treatment

The conditioned medium secreted by space-flown cells, otherwise known as secretome, was collected from the cell chambers separately from the cells. The samples were placed in numbered tubes and subsequently frozen at −80 °C. Environmental parameters and the experimental timeline for the 1G NSCs were based on that of the ISS in order to simulate the conditions of the space flight. Upon its recovery, we mixed two parts of culture medium per one part of straight secretome to treat naïve NSCs grown in 1G cells. The secretome was used straight (as it was obtained from the devices); no concentration was necessary to see the effects shown in this paper. The 1G naïve sister cells of the space-flown cells were first imaged with basal stem cell medium. The medium was switched to fresh medium containing secretome at 44.5 h into the time-lapse video.

### 4.3. Time-Lapse Microscopy Analysis

Asynchronous experiments were performed following spaceflight on Earth for the study of cell proliferation. The time-lapse microscopy imaging was conducted using the ZEISS Axio Observer 7 fully motorized inverted research microscope equipped with Definite Focus 2, ZEISS Axiocam 506 monochrome camera with ZEISS ZEN software, and ZEISS Full Incubation XL chamber (Oberkochen, Germany) for temperature and CO_2_ control with a motorized scanning stage. The microscope collected images every 15 min in seven different XYZ positions for a duration of about 72 h for each cell condition. The time-lapse data were analyzed frame-by-frame by hand to scan for instances of ACD including ICD and MDCD with the ZEN Blue 3.5 program. All data were charted in Microsoft Excel for statistical analysis.

### 4.4. Immunofluorescence and Nuclear Staining

**Figure 10:**
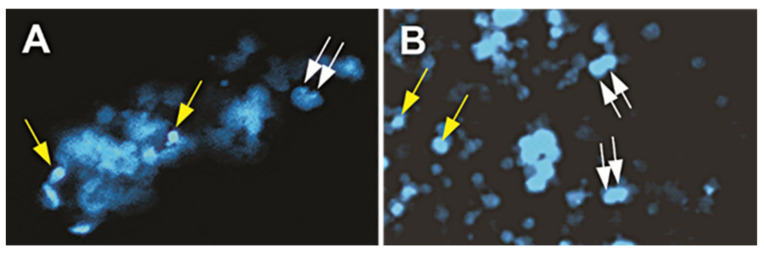
Representative views of NucBlue used to stain cell nuclei to study nuclear abnormalities. Dividing cells are indicated with white arrows and normal single nuclei cells are indicated with yellow arrows. (**A**) 1G cells with NucBlue stain faintly expressed in nuclei. (**B**) SPC-µG exposed cells two weeks post-spaceflight with NucBlue stain faintly expressed in nuclei.

Double immunofluorescence using established markers for NSCs was performed with the following antibodies used at a 1:100 dilution of nestin (BD Pharmingen, Franklin Lakes, NJ, USA) and tubulin. NucBlue™, (Gibco|Thermo Fisher Scientific, Waltham, MA, USA) was also used for staining the nuclei of the NSCs (Figure 10). Immunofluorescence procedures included plating cells onto either coated plastic 8-well chambers (Nunc 177445), or poly-d-lysine coated glass coverslips. To visualize the markers, the secondary antibodies used were goat anti-rabbit IgG Texas Red (JIR) and goat anti-mouse IgG FITC (Sigma, Tokyo, Japan). 

## Data Availability

All data have been included in the manuscript.

## Supplementary Materials

The following supporting information can be downloaded at: https://www.mdpi.com/article/10.3390/ijms232214320/s1.
